# Differentiation, functions, and roles of T follicular regulatory cells in autoimmune diseases

**DOI:** 10.1186/s41232-021-00164-9

**Published:** 2021-05-03

**Authors:** He Hao, Shingo Nakayamada, Yoshiya Tanaka

**Affiliations:** 1 First Department of Internal Medicine, School of Medicine, University of Occupational and Environmental Health, Japan, 1-1 Iseigaoka, Yahata-nishi, Kitakyushu, 807-8555 Japan; 2 Department of Immuno-oncology, The Fourth Hospital of Hebei Medical University, Shijiazhuang, China

**Keywords:** T follicular regulatory cells, Differentiation, T follicular helper cells, Therapeutic targets, Autoimmune diseases

## Abstract

T follicular helper cells participate in stimulating germinal center (GC) formation and supporting B cell differentiation and autoantibody production. However, T follicular regulatory (Tfr) cells suppress B cell activation. Since changes in the number and functions of Tfr cells lead to dysregulated GC reaction and autoantibody response, targeting Tfr cells may benefit the treatment of autoimmune diseases. Differentiation of Tfr cells is a multistage and multifactorial process with various positive and negative regulators. Therefore, understanding the signals regulating Tfr cell generation is crucial for the development of targeted therapies. In this review, we discuss recent studies that have elucidated the roles of Tfr cells in autoimmune diseases and investigated the modulators of Tfr cell differentiation. Additionally, potential immunotherapies targeting Tfr cells are highlighted.

## Background

Autoimmune diseases are characterized by immune system dysregulation, which mounts immune responses against autoantigens, leading to severe inflammation in various tissues and organs [1]. Even though traditional therapies, such as immunosuppressants and corticosteroids, have improved the survival rate of patients with autoimmune diseases, some patients are non-responsive or resistant to these drugs. Therefore, the development of innovative therapeutics that can restore the immune system is urgently required.

T follicular helper (Tfh) cells are a heterogeneous subset of CD4^+^ T cells that participate in stimulating germinal center (GC) formation, supporting B cell survival, differentiation, and proliferation [2]. Tfh cells are characterized by the expression of receptor chemokine (C-X-C motif) receptor 5 (CXCR5), transcriptional repressor B cell lymphoma 6 (Bcl-6), programmed cell death protein-1 (PD-1), inducible T cell co-stimulator (ICOS), and interleukin (IL)-21 [3]. Dysregulation of Tfh cells is associated with the pathogenesis of a number of autoimmune diseases, including systemic lupus erythematosus (SLE), rheumatoid arthritis (RA), Sjögren’s syndrome, idiopathic inflammatory myopathies, Graves’ disease, Hashimoto’s disease, and type I diabetes [3].

Recently, a subpopulation of T regulatory (Treg) cells named T follicular regulatory (Tfr) cells that co-express markers of both Treg and Tfh cells has been identified [4–7]. Apart from expressing Tfh-related markers, Tfr cells also express regulatory markers, such as forkhead box p3 (Foxp3), CD25, cytotoxic T-lymphocyte-associated protein 4 (CTLA-4), IL-10, and transforming growth factor-beta (TGF-β) [4, 8, 9]. Tfr cells exhibit different phenotypic characteristics at different stages of maturation. For example, a study identified CD25^−^ Tfr cells with inhibitive functions in the tonsils [10].

Tfr cells are involved in a specialized immune regulation of antibody maturation and germinal center formation via interactions with Tfh and/or B cells [4, 6, 9]. Recent studies have reported that Tfr cell dysregulation contributes to the accumulation of autoantibodies, leading to a wide range of autoimmune diseases [11]. Thus, targeting Tfr cells may be a useful strategy to treat autoimmune diseases. In this review, we discuss recent discoveries about Tfr cells, especially the mechanism of Tfr cell differentiation.

## Roles of Tfr cells in inflammation and immunity

Several studies have demonstrated that Tfr cells restrict Tfh-cell activity and GC reaction, suppressing autoantibody expansion [12]. The regulatory mechanism of Tfr cells mainly depends on co-inhibitory receptors, inhibitory cytokines, and granzyme release. For example, CTLA-4 acts as a direct suppressor of Tfr cells. CTLA-4 can limit the expression of co-stimulatory ligands CD80 and CD86 on B cells and control the overreaction of GCs [13–15]. TGF-β, which is highly produced by Tfr cells, critically inhibits Tfh cell expansion, self-reactive B cell activation in the GC, and autoantibody production [16]. Additionally, exosomal TGF-β from hepatocytes infected with the hepatitis C virus promotes Tfr cell expansion and suppresses Tfh-cell functions [17].

However, a few recent studies have elucidated that Tfr cells can promote B cell growth in the GC. IL-10 from the precursor B cells in the marginal zone is required for the differentiation and positioning of Tfr cells in the secondary lymphoid tissues [18]. Previous studies have suggested that Tfr cells enhance the formation of IL-10–producing B cells or B regulatory cells [19, 20], thereby controlling aberrant GC responses. In contrast, IL-10 produced by Tfr cells can promote B cell differentiation and GC development not only in food allergy immune responses but also in acute infections with lymphocytic choriomeningitis virus [21, 22]. Notably, Tfr cells repress the development of granzyme-B–expressing cytotoxic Tfh cells and promote GC responses and production of high-affinity autoantibodies [23]. The above findings suggest that Tfr cells behave differently in different environments.

## Modulators of Tfr cell differentiation

**Fig. 1:**
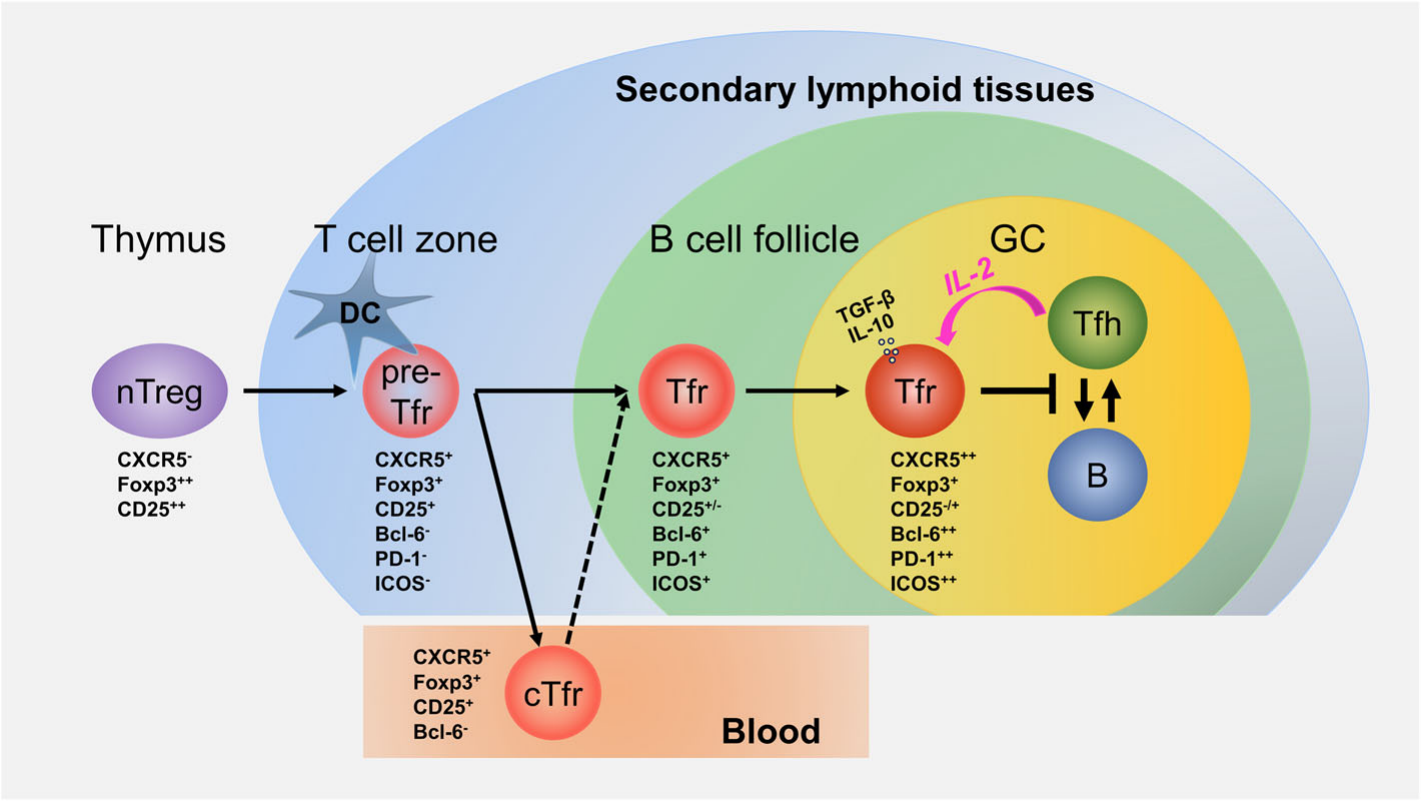
Model for T follicular regulatory (Tfr)-cell differentiation. Pre-Tfr cells are generated in the T cell zone where thymic natural Treg cells interact with dendritic cells. Pre-Tfr cells migrate into the B cell follicle, strengthening the Tfr cell transcriptional program. Alternatively, pre-Tfr cells can exit the secondary lymphoid tissues and enter the peripheral blood as circulating Tfr (cTfr) cells. In the germinal center (GC), Tfr cells terminally differentiate into mature Tfr cells, and this process fine-tunes Tfh and B cells. cTfr cells may migrate into follicles and the GC after reactivation. Furthermore, interleukin (IL)-2 might facilitate Tfh cell conversion into Tfr cells in the GC. Markers involved in Tfr cell differentiation are presented in black font

Tfr cells are located in the secondary lymphoid organs, including the spleen, lymph nodes, Peyer’s patches, and peripheral blood. Unlike Tfh-cell differentiation, which originates directly from naive CD4^+^ T cells or already differentiated effector T cells [8, 24], Tfr cell differentiation is thought to arise from thymus-derived Treg cells or naive Foxp3^−^ precursors in a programmed death-1 ligand (PD-L1)-dependent manner [4, 6, 25]. During the initial stage, pre-Tfr cells are derived from CXCR5^**-**^Foxp3^+^ thymic natural Treg cells upon interaction with dendritic cells (DCs) [26] (Fig. 1). After priming with DCs, pre-Tfr cells can enter the peripheral blood or migrate to the B cell follicle [26]. The generation of circulating Tfr (cTfr) cells requires priming by DCs but does not require B cell interactions [27]. Furthermore, cTfr cells may move back to the follicles and GC post-reactivation [9]. In the B cell follicle, Tfr cells strengthen the transcriptional program following the upregulation of Bcl-6, ICOS, and PD-1 after interaction with follicle B cells [26]. At the final maturation process, Tfr cells move to the GC and terminally differentiate into mature Tfr cells, which can suppress Tfh and GC B cells [26]. Additionally, Tfr cells can be generated from the reprogramming of Tfh cells through IL-2 stimulation in vitro [28], indicating the conversion of Tfh cells into Tfr cells by IL-2 in the GC.

Many positive and negative regulators are involved in Tfr cell differentiation. Understanding the signals regulating Tfr cell generation is vital for the development of targeted therapies. Therefore, we discuss co-stimulator molecules, cytokines, transcription factors, and noncoding-RNAs that can act as modulators of Tfr cell differentiation.

### Co-stimulatory molecules

**Fig. 2:**
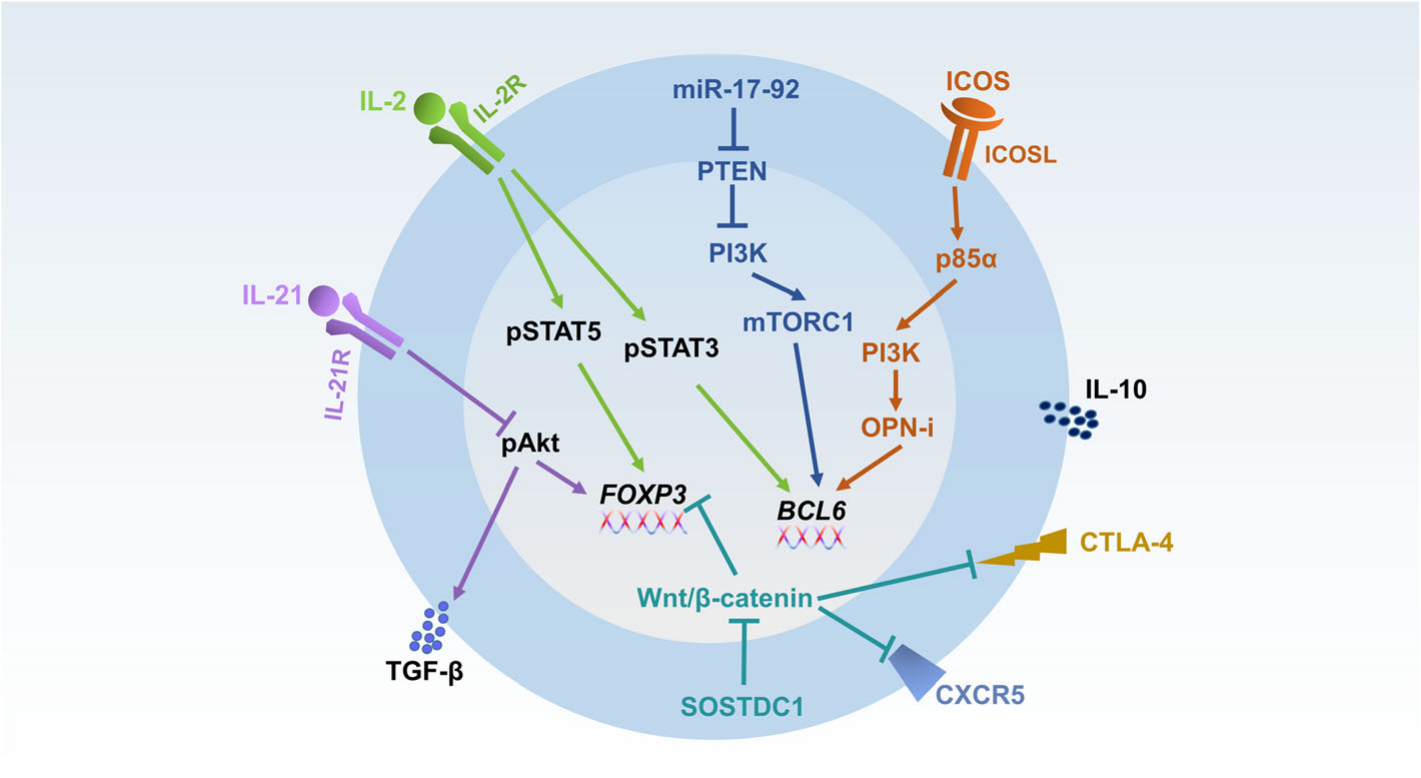
Representative signaling modulation of Tfr cell differentiation. The ICOS signaling activates the p85α-OPN-i axis and promotes Bcl-6–dependent Tfr cell differentiation. IL-2–activated STAT3 and STAT5 selectively bind to *FOXP3* and *BCL6* genes, respectively, thereby converting memory Tfh cells to functional Tfr cells. IL-21 inhibits Tfr cell development by blocking the pAkt signaling, thereby reducing the expression of TGF-β and Foxp3. SOSTDC1 enhances Tfr cell differentiation and function by blocking the Wnt/β-catenin axis and subsequently upregulating Foxp3, CXCR5, and CTLA-4. miR-17-92 promotes Tfr cell differentiation by enhancing the PI3K-mTORC1 signaling.

Tfr cell development requires T cell receptor and co-stimulatory signals through CD28 and ICOS [4, 28, 29]. In CD28- or ICOS-deficient mouse models, Tfr cells are attenuated in the lymph nodes [28, 29]. The specific mechanism of Tfr cell development reportedly involves the ICOS signaling, which transiently inactivates forkhead/winged-helix transcription factor 1, whereby Bcl-6 is upregulated, and Tfr cell differentiation is promoted [30]. Additionally, ICOS activation promotes Bcl-6–dependent Tfr cell differentiation by enhancing the interaction between intracellular osteopontin (OPN-i) and p85α, and this interaction plays a critical role in regulating phosphoinositide 3-kinases (PI3K) activity in mice [31] (Fig. 2). OX40L promotes Tfh-cell differentiation but blocks Treg-cell functions in both mice and humans [32, 33]. The suppressive ability of Tfr cells is impaired when they are exposed to soluble OX40L [34]. Moreover, glucocorticoid-induced TNF receptor family related protein skews the Tfh/Tfr ratio toward Tfh cells and upregulates IL-21, suppressing Tfr cell proliferation [35]. A case report has shown that cTfh and cTfr cells are severely compromised in a child with CD40 deficiency, indicating that CD40 is required for Tfh- and Tfr cell development [36]. However, CD40-CD40L interactions render resistance to Tfr-mediated suppression of Tfh-induced B cell proliferation [37].

The impact of PD-1/PD-L1 signaling in the differentiation of Tfr cells is complex. Both Tfh and Tfr cells show high levels of PD-1 expression. PD-1 inhibits the differentiation and functioning of Tfr cells, and PD-1–deficient Tfr cells potently control antibody production in mouse models [29]. PD-L1 promotes the generation of Tfr cells from Foxp3-producing precursors cells after immunization [38]. Conversely, PD-L1 blockage supports Tfr cell accumulation in an allogeneic normal pregnant mouse model [39]. CTLA-4 is also highly expressed in Tfr cells [13]. Deletion of CTLA-4 increases the frequency and an absolute number of Tfr cells but leads to a defective suppression of antigen-specific antibody responses in the effector phase [13–15].

### Cytokines

Tfh cells are repressed by IL-2 through the signal transducer and activator of transcription 5 (STAT5)-dependent suppression of Bcl-6 [40–43]. However, the role of IL-2 in Tfr cell differentiation is inconsistent. High concentrations of IL-2 at the peak of influenza infection facilitate Tfr cell development. However, as the infection resolves, IL-2 levels decrease, and IL-2Rα^hi^ Treg cells differentiate into mature GC Tfr cells by downregulating IL-2Rα and upregulating Bcl-6 [44]. IL-2 dynamically regulates Tfr cell responses, and low IL-2 levels may be required to induce Tfr cells. In a human study, exogenous IL-2–activated STAT3 and STAT5 selectively bind to *FOXP3* and *BCL6* gene loci, respectively, thereby converting memory Tfh cells to functional Tfr cells [28] (Fig. 2). Moreover, IL-2 positively influences Tfr cell differentiation in the GC in vitro [45–47].

Tfr cells suppress Tfh-induced B cell proliferation by inhibiting IL-21 production [37]. IL-21 restrains Tfr cell development by reducing the phosphorylation of protein kinase B (Akt) and consequently suppresses Foxp3 and TGF-β production in mice [48] (Fig. 2). Additionally, IL-21 can stimulate the Tfr cell–suppressed B cell metabolism by enhancing glycolysis [49]. IL-21/IL-21R interaction inhibited Tfr cell development through Bcl-6 upregulation, constraining IL-2Rα (CD25) expression in mouse models [50]. Similarly, IL-6 may also affect Tfr cell development by preventing the binding of STAT5 to the *IL2rb* locus [41].

### Transcription factors

Foxp3 and Bcl-6 are the master transcriptional factors for Tfr cell differentiation. Defects in the Bcl-6–nucleosome remodeling and deacetylase complex impair Tfr cell development [51]. Foxp3, along with the chromatin-modifying enzyme enhancer of zeste homolog 2, possesses the capacity to convert Tfh cells into functional suppressive Tfr-like cells [27]. B lymphocyte maturation protein 1 (Blimp-1) suppresses Tfh-cell differentiation by reducing Bcl-6 expression [52, 53]. Blimp-1 can lead to Tfr cell homing into the GC via CXCR5 and CCR7 expression regulation [54]. Additionally, Tfr cell-derived IL-10 is also controlled by Blimp-1 [21]. Notably, Blimp-1 loss doubles the Tfr population but reduces the suppressive ability of Tfr cells [55].

The nuclear factor of activated T cell, cytoplasmic 1 (NFATc1), is essential to enhance the expression of the chemokine CXCR5 on Tfr cells, whereas NFATc1-deficient Tfr cells cause augmented GC reactions [56]. Store-operated Ca2^+^ entry can control NFATc1-mediated Tfr cell differentiation via the expression of interferon regulatory factor 4, basic leucine zipper atf-like transcription factor, and Bcl-6 [57]. C-Musculoaponeurotic fibrosarcoma (c-Maf) promotes the expression of IL-21 and CXCR5 [58, 59], and deletion of c-Maf leads to a striking Tfr cell loss [60]. Additionally, inhibitors of DNA binding 2 (Id2) and Id3 directly upregulate Tfr-associated genes, such as IL-10 and CXCR5 [61]. Interestingly, the high expression of Id2 or Id3 strengthens the early Tfr cell checkpoint, whereas during the intermediate developmental phases, the low abundance of Id2 and Id3 ensures the development of fully mature Tfr cells [61].

Nuclear factor-kappa B (NF-κB) plays a vital role in regulating immune responses [62]. Patients with *NF-κB2* mutations have reduced Tfh and Tfr cell counts [63]. The tumor necrosis factor receptor-associated factor 3 signaling maintains a high ICOS expression in Treg cells, and a high ICOS level is required for Tfr cell generation [64]. Moreover, in mice, sclerostin domain-containing protein 1, secreted by a Tfh cell subpopulation, facilitates Tfr cell differentiation by blocking the Wnt/β-catenin axis, upregulating Foxp3, CXCR5, and CTLA-4 expression [65] (Fig. 2).

### Noncoding-RNAs

miR-17-92 overexpression leads to increased Tfh and Tfr cell frequencies in a viral infection mouse model [66]. Additionally, in mice, miR-17-92 promotes Tfr cell differentiation by suppressing the PI3K inhibitor phosphatase and tensin homolog (PTEN) expression and by enhancing the PI3K-Akt-mTOR signaling [67] (Fig. 2). Another study has shown that miR-17-92 deficiency in T cells increases the number of Tfr cells in a mouse model of chronic graft versus host disease. Thus, miR-17-92 might regulate Tfr cell development depending on the experimental models [68]. miR-155 maintains the competitive fitness of Foxp3^+^ regulatory T cells by targeting the suppressor of cytokine signaling (SOCS1), an inhibitory signaling pathway for the IL-2/STAT5 signaling pathway [69]. However, miR-155 overexpression leads to a lack of Tfr cells and enhances the B cell activation in the GC [70].

miR-146a deficiency in T cells promotes Tfr cell expansion by enhancing the ICOS signaling, suggesting that miR-146a inhibits Tfr cell differentiation [71]. Tfr cells express a higher level of miR-10a than Treg cells, and it has been proposed that miR-10a stimulates Treg cell conversion to Tfr cells via Bcl-6 expression regulation [72]. Moreover, blocking miR-124 expression enhances Tfr cell differentiation and increases IL-10 production [73].

## Pathological significance of Tfr cells in autoimmune diseases

Tfr cells have been assessed in a wide range of autoimmune diseases. Since it is challenging to obtain Tfr cells from human lymphoid tissues, cTfr cells have been evaluated as an alternative population to assess GC reaction in most studies. Functional impairment and altered proportion of cTfr cells are associated with autoimmune diseases, such as RA, SLE, systemic sclerosis, ankylosing spondylitis, and IgG4-related disease [11]. Interestingly, coronavirus disease 2019 (COVID-19) convalescent patients with a severe disease showed a higher percentage of effector-memory-like cTfh cells but a lower percentage of cTfr cells compared with healthy donors [74].

As Tfh and Tfr cells play opposing regulatory roles in GC responses, an imbalance in their actions may eventually promote the development of autoimmune diseases. Indeed, a disrupted balance of Tfh and Tfr cells has been related to various autoimmune diseases, including RA, SLE, SSc, multiple sclerosis, ulcerative colitis, and autoimmune hepatitis [75–81]. The percentage of activated cTfr cells and activated Tfr/Tfh cell ratio are significantly decreased in SLE patients than in healthy donors, and active patients have a lower ratio of activated Tfr/Tfh cells than inactive patients [28]. Taken together, these results suggest that Tfr cells can be used as novel biomarkers and potential therapeutic targets for autoimmune diseases.

## Targeting Tfr cells for the treatment of autoimmune diseases

Tfr cells play a prominent role in the pathogenesis of autoimmune diseases. Current therapeutic drugs work by altering Tfr cell differentiation or function. In RA patients, the percentages of cTfh and cTfr are significantly reduced after methotrexate treatment [82]. cTfr cell counts are decreased in type 1 diabetes, and this deficiency in cTfr cells can be rescued by rituximab, an anti-CD20 monoclonal antibody [83]. Abatacept, a CTLA-4-Ig fusion protein that binds to CD80 and CD86, decreases the proportions of cTfh and cTfr cells in patients with relapsing-remitting multiple sclerosis [84]. Furthermore, in patients with seasonal allergic rhinitis, cTfr cells are induced following grass-pollen subcutaneous immunotherapy or sublingual immunotherapy accompanied by an alteration in the epigenetic landscape [85]. Additionally, the number of cTfr cells is decreased in patients with allergic rhinitis, and the frequency and functions of cTfr cells are recovered after allergen immunotherapy [86].

Our recent study analyzed the influence of treatment status on Tfh and Tfr cell proportions in SLE patients [28]. No significant correlation was observed between the dose of corticosteroid and the numbers of activated peripheral Tfh and Tfr cells. Furthermore, immunosuppressants reduce the percentages of activated Tfh cells but not activated Tfr cells [28]. Similar to our results, in patients with antineutrophil cytoplasmic antibody-associated vasculitis, the remission achieved through immunosuppressive treatment decreases Tfh cell percentage, but no significant alterations have been observed in Tfr cells [87].

**Fig. 3:**
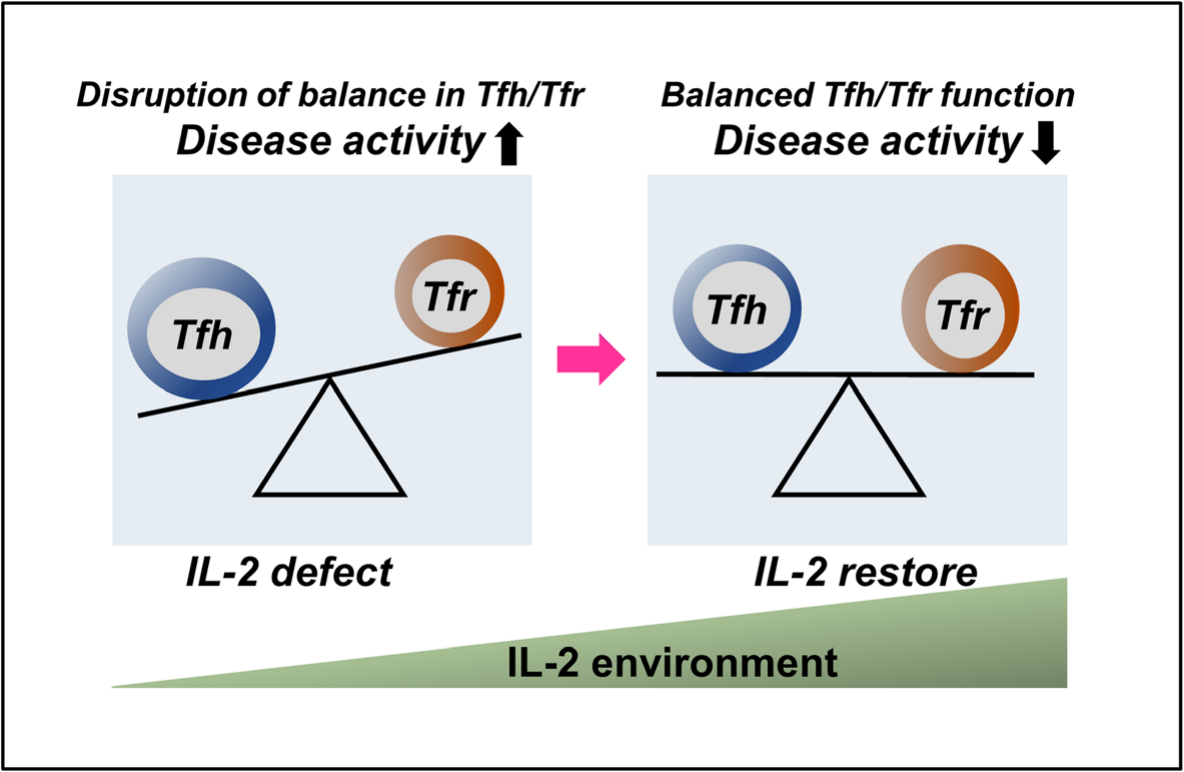
Importance of IL-2 in Tfh/Tfr cell balance. The imbalance of Tfh/Tfr cells leads to defective IL-2 production. Exogenous IL-2 restores the balance between Tfh and Tfr cells, thus offering therapeutic approaches

Proof-of-concept clinical trials have shown the efficacy of low-dose IL-2 treatment via selectively expanding Treg cells and inhibiting Th17 and Tfh cell proliferation in SLE patients [88, 89]. However, Tfr cells have not been explored yet. In an in vitro experiment, we discovered that a low-dose of IL-2 (10 ng/mL) restores Tfr cell functions by directly promoting activated Tfr cells and indirectly through Tfh cell reprogramming to Tfr cells via STAT3 and STAT5 activation. Fine-tuning the balance between Tfh and Tfr cells might provide therapeutic approaches in SLE (Fig. 3). Furthermore, the Treg-specific IL-2 therapy is under investigation; it will foreseeably help improve and establish low-dose IL-2 therapies [90–92].

## Conclusion

The discovery of Tfr cells shed new light on the regulation of GC formation and B cell maturation. Analyses of Tfr cells reveal that they may serve as a potential biomarker for disease severity, and targeted therapeutic approaches for the treatment of autoimmune diseases can be developed accordingly. Differentiation of Tfr cells requires various closely linked regulators, which play synergistic or antagonistic roles in Tfr cell differentiation. Further studies are required to clarify the manipulations of the molecular network regulating Tfr cell formation and physiology. Moreover, detailed studies on Tfr cells and clinical trials are needed to understand the immunotherapeutic efficacy of targeting Tfr cells.

## Data Availability

No additional data are available.

## Abbreviations

Tfh: T follicular helper
GC: Germinal center
Tfr: T follicular regulatory
CXCR5: Chemokine receptor chemokine (C-X-C motif) receptor 5
CXCL13: C-X-C motif chemokine ligand 13
PD-1: Programmed death-1
ICOS: Inducible T cell co-stimulator
CTLA-4: Cytotoxic T-lymphocyte-associated protein 4
TGF-β: Transforming growth factor-beta
RA: Rheumatoid arthritis
SLE: Systemic lupus erythematosus
SSc: Systemic sclerosis
OPN-i: Intracellular osteopontin
Blimp-1: B lymphocyte maturation protein 1
NFAT: Nuclear factor of activated T cell
NF-κB: Nuclear factor kappa B
Id: Inhibitor of DNA binding
PI3K: Phosphoinositide 3-kinases
IL: Interleukin
Treg: T regulatory
Bcl-6: B cell lymphoma 6
Foxp3: Forkhead box p3
DC: Dendritic cell
cTfr: Circulating Tfr
PD-L1: Programmed death-1 ligand
STAT: Signal transducer and activator of transcription
Akt: Protein kinase B
c-Maf: C-musculoaponeurotic fibrosarcoma
